# Transcriptomic and functional analyses of the piRNA pathway in the Chagas disease vector *Rhodnius prolixus*

**DOI:** 10.1371/journal.pntd.0006760

**Published:** 2018-10-10

**Authors:** Tarcisio Brito, Alison Julio, Mateus Berni, Lisiane de Castro Poncio, Emerson Soares Bernardes, Helena Araujo, Michael Sammeth, Attilio Pane

**Affiliations:** 1 Institute of Biomedical Sciences (ICB), Federal University of Rio de Janeiro, Rio de Janeiro, Brazil; 2 Institute of Molecular Entomology (INCT), Rio de Janeiro, Brazil; 3 Institute of Biophysics Carlos Chagas Filho (IBCCF), Federal University of Rio de Janeiro, Rio de Janeiro, Brazil; 4 Forrest Brasil Tecnologia Ltda, Araucária, Paraná, Brazil; 5 Nuclear Energy Research Institute, Radiopharmacy Center, São Paulo, Brazil; University of California Davis, UNITED STATES

## Abstract

The piRNA pathway is a surveillance system that guarantees oogenesis and adult fertility in a range of animal species. The pathway is centered on PIWI clade Argonaute proteins and the associated small non-coding RNAs termed piRNAs. In this study, we set to investigate the evolutionary conservation of the piRNA pathway in the hemimetabolous insect *Rhodnius prolixus*. Our transcriptome profiling reveals that core components of the pathway are expressed during previtellogenic stages of oogenesis. *Rhodnius*’ genome harbors four putative *piwi* orthologs. We show that *Rp-piwi2*, *Rp-piwi3* and *Rp-ago3*, but not *Rp-piwi1* transcripts are produced in the germline tissues and maternally deposited in the mature eggs. Consistent with a role in *Rhodnius* oogenesis, parental RNAi against the *Rp-piwi2*, *Rp-piwi3* and *Rp-ago3* results in severe egg laying and female adult fertility defects. Furthermore, we show that the reduction of the *Rp-piwi2* levels by parental RNAi disrupts oogenesis by causing a dramatic loss of trophocytes, egg chamber degeneration and oogenesis arrest. Intriguingly, the putative Rp-Piwi2 protein features a polyglutamine tract at its N-terminal region, which is conserved in PIWI proteins encoded in the genome of other Triatomine species. Together with *R*. *prolixus*, these hematophagous insects are primary vectors of the Chagas disease. Thus, our data shed more light on the evolution of the piRNA pathway and provide a framework for the development of new control strategies for Chagas disease insect vectors.

## Author summary

*Rhodnius prolixus* together with other blood-feeding bugs of the Triatominae family are primary vectors of the protozoan *Trypanosoma cruzi*, the causative agent of the Chagas disease. It has been estimated that 7–8 million people are affected by this life-threatening illness worldwide, which makes the Chagas disease one of the most neglected tropical diseases. In this study, we describe the transcriptome of previtellogenic stages of *Rhodnius* oogenesis. Furthermore, by using a combination of molecular biology techniques and functional analyses we show that central components of the piRNA pathway are conserved in this species. The piRNA pathway guarantees genomic stability in the germ cells of organisms as distant as flies and mice. In accordance, we find that the knock-down of the *piwi* genes, which form the backbone of the pathway, results in partial or complete female adult sterility in *Rhodnius*. Our data will help improve the annotation of the *Rhodnius* genome and provide a framework for the development of novel techniques aiming at the eradication of *Rhodnius prolixus* and other Triatomine species from the infested areas. The achievement of this goal will ultimately prevent the transmission of trypanosomes to humans and reduce or eliminate the diffusion of the Chagas disease.

## Introduction

PIWI-clade Argonaute proteins have been implicated in a range of cellular and developmental events by regulating gene expression and imposing transposon silencing [1–3]. These proteins appear to be particularly critical for the maintenance of genomic stability during gametogenesis in a range of animal species including flies, worms and mice. The activity of the PIWIs is generally associated with the biogenesis and function of a specific class of 23-30nt small non-coding RNAs termed Piwi-interacting RNAs or piRNAs [4,5]. The details of the piRNA pathway have been mostly elucidated using the ovary of the fruit fly *Drosophila melanogaster* as model system. In this species, two branches of the pathway were shown to act in the germline and in the somatic follicle cells respectively. *Drosophila* harbors three PIWI proteins: Piwi, the founding member of this protein family, is mostly nuclear and acts in both arms of the piRNA pathway [4,6], while Aubergine (Aub) and Argonaute3 (Ago3) are expressed exclusively in the germ cells [7]. PIWI proteins belong to the Argonaute family and are characterized by typical PAZ, MID and Piwi domains. The PAZ and the MID domains interact with the mature piRNAs, which provide target specificity to the RNAse H slicing activity harbored in the Piwi domain.

In *Drosophila*, piRNA precursor transcripts (i.e. pre-piRNAs) are mostly transcribed from genomic regions densely populated by transposon remnants and known as piRNA clusters[4]. A second source of piRNAs is provided by the transcripts generated by active transposable elements dispersed in the genome. The RDC complex, which is formed by the Cutoff (Cuff), Rhino (Rhi) and Deadlock (Del) proteins, together with the transcription factor Moonshiner (Moon) and components of the THEO/Trex complex orchestrate that transcription of the piRNA clusters in the germline tissues and the transport of the pre-piRNAs from the nucleus to the cytoplasm [8–13]. The nuclei of *Drosophila* germ cells are surrounded by a membraneless organelle called the nuage, which hosts several enzymatic activities including the PIWI proteins Aub and Ago3, the DEAD-box helicase Vasa (Vas), and the tudor domain proteins Tudor, Krimper, Tejas and Papi [14]. These proteins act at different levels to process the pre-piRNAs and produce the mature 23-30nt long piRNAs. A critical role in the biogenesis of the piRNAs is exerted by Aub and Ago3, which engage in feedforward amplification mechanisms termed the ping-pong cycle [4,15]. The ping-pong cycle amplifies the piRNA population and couples piRNA biogenesis with the degradation and, consequently, with the downregulation of transposon transcripts. Recent studies revealed that the Vas protein, a well conserved germline-specific DEAD-box helicase, provides an essential scaffold to anchor the Aub protein and to promote piRNA production [16]. Finally, antisense piRNAs are bound by Piwi, that translocates into the nucleus and employs the piRNAs as guide to locate and silence active transposable elements [17]. The second branch of the pathway acts in the somatic cells of the ovary. Slicing of the precursor transcripts from somatic piRNA clusters generates antisense piRNAs, which guide Piwi to silence transposons of the *zam*, *gypsy* and *idefix* families. piRNA biogenesis requires the Zucchini (Zuc) endonuclease, the helicase Armitage (Armi) and the Tudor domain proteins YB, SoYB, BoYB, Vreteno and T2RD2, which accumulate in a cytoplasmic organelle known as the YB-body [18,19]. Both in the somatic as well as in the germline tissues of the fly ovary, the piRNA pathway protects the cells from the deleterious effects of massive transposon mobilization [2,20,21].

In *Drosophila*, mutations in PIWI proteins result in complete female adult sterility. Consistent with Aub and Ago3 being restricted to germline tissues, the absence of these factors culminates in a severe loss of stem and germ cells, a failure to assemble the chromatin in the oocyte nucleus (i.e. the karyosome phenotype) and the disruption of the dorsal-ventral polarity of the egg chamber and the future embryo [22–25]. Mutations in Piwi instead affect both the development of the follicular epithelium and of the germline. Also, this protein appears to act both in concert with or independent of the piRNAs [21].

A variable number of *piwi* genes originating from duplication events has been reported in various insect species. Among the Hemipteran insects, *Rhodnius prolixus*’ genome harbors 3 *piwi* genes and one ortholog of *ago3*, while 8 *piwis* and 2 copies of the *ago3* gene were observed in the aphid *Acyrthosiphon pisum* [26]. Thus, in various animals *piwi* undergoes gene amplification, and the different copies often display stage- and tissue-specific expression patterns suggesting functional specialization. For instance, the mosquito *Aedes aegypti* harbors 7 *piwi* orthologs, whereby the Piwi5 and Ago3 proteins have been connected to the biogenesis of viral piRNAs [27]. piRNAs and *piwi* orthologs have been also identified in mosquitos of the Anopheles genus [28,29]. *piwi* and *vas* have been extensively used to investigate the segregation of germ cell determinants in different species [30]. However, functional studies addressing the role of Piwi genes in insects other than *Drosophila* are still scarce.

The blood-feeding insect *Rhodnius prolixus* is a primary vector of *Trypanosoma cruzi*, the etiologic agent of the Chagas disease [31]. The Chagas disease is life-threatening illness that currently affects 7–8 million people worldwide. Despite its medical relevance, the molecular events that drive oogenesis and guarantee adult fertility in *Rhodnius* are largely unknown. In this study, we employed transcriptome profiling to unveil the genetic and molecular basis underlying *Rhodnius* oogenesis. Our results reveal that central components of the piRNA pathway are conserved in this species and are expressed early during oogenesis. Furthermore, we show that *Rp-piwi2*, *Rp-piwi3* and *Rp-ago3*, but not *Rp-piwi1*, are expressed in *Rhodnius* ovaries, accumulate in germline tissues and are necessary for female adult fertility.

## Materials and methods

### *Rhodnius* handling and whole-mount immunostaining

*Rhodnius* females were dissected 10 days after the feeding regimen and ovaries were immediately placed in cold Phosphate Buffered Saline (PBS). Ovaries were fixed and immunostained as previously described [32]. The anti-γH2Ax (Millipore) and DAPI were diluted 1:1000 in PBS + Tween20 0.3% supplemented with 1% BSA. Ovaries were mounted in 70% glycerol and analyzed on a Leica Confocal Microscope.

### Phylogenetic construction

The evolutionary history of PIWI proteins in *Rhodnius* and *Drosophila* was inferred applying a Maximum Likelihood method [33]. The analysis included a total of seven amino acid sequences, which were aligned by the Multiple Sequence Alignment with Log Expectation (MUSCLE, version 3.8.31) method [34], employing standard parameters. The evolutionary history was inferred by Molecular Evolutionary Genetics Analysis version 6.0 (MEGA6), and visualized using interactive Tree of Life (iTOL, v2). The tree was validated by 1000 bootstraps replications. Values higher than 90% were indicated in nodes. The amino acid alignments performed to highlight the Rp-Piwi2 PolyQ stretch in Triatominae species included the following sequences: JAI55027.1 (*R*. *neglectus*), JAP02788.1 (*T*. *dimidiata*), JAC16725.1 (*T*. *infestans*) available in NCBI, and Rp-Piwi2 of *R*. *prolixus*. The aminoacid sequences of proteins from *R*. *prolixus* and *D*. *melanogaster* were obtained from VectorBase (https://www.vectorbase.org/) and FlyBase (http://flybase.org/) respectively.

### Total RNA extraction and RT-PCR assays

For the RT-PCR assays, total RNA was extracted from previtellogenic stages and, separately, from choriogenic stages dissected from 10/15 adult females. For qRT-PCR assays, total RNA was extracted from previtellogenic stages of wildtype, pRNAi and control ovaries 2 weeks after blood feeding. Tissues were ground in Trizol Reagent (Invitrogen) and processed as per manufacturer instructions. Total RNA was treated with Turbo DNA-free (Ambion) to remove genomic DNA traces. The resulting DNA-free total RNA was subjected to *in vitro* Reverse Transcription (RT) with Superscript III (Invitrogen). 1.0μg of DNA-free total RNA was used for each reaction and assays were conducted in biological triplicates. The oligonucleotides used in RT-PCR and qRT-PCR assays, are listed in the S1 Table.

### Parental RNAi and *in situ* RNA hybridization assays

For *in vitro* transcription, T7 promoter sequences in the appropriate orientation were added to the DNA templates through a PCR-based system using the set of oligonucleotides listed in S1 Table. The same DNA templates were adopted both for *in situ* hybridization assays and for dsRNA production. For parental RNAi assays, sense and antisense ssRNAs for each gene were produced using the Megascript kit (Ambion). Approximately equal amounts of sense and antisense RNAs for each target were mixed in annealing buffer, precipitated and resuspended in water to a final concentration of approximately 1.5μg/μL. Two microliters of each dsRNA were injected in the abdomen of adult females three days prior blood feeding. A total of 10 adult females were injected with each dsRNA preparation. Ovaries were dissected two weeks after the feeding regimen. The DNA templates used to generate *in situ* hybridization probes were obtained by PCR using oligonucleotides carrying T7 promoter sequences at the 5'-end. The templates were subjected to in vitro transcription with the DIG RNA labeling kit (Roche). *In situ* hybridization conditions have been described elsewhere [35]. Oligonucleotides used in this study are listed in the S1 Table.

### RNAseq library preparation and bioinformatic analyses

Ovaries of 10 blood-fed *Rhodnius* females were dissected in cold PBS and previtellogenic stages of oogenesis were manually separated from vitellogenic stages and chorionated eggs. Total RNA from two biological replicates was isolated with Trizol reagent (Invitrogen) and subjected to paired-end RNA library preparation as per manufacturer's instructions (Illumina, Truseq paired-end RNA library prep. kit). The libraries were sequenced on Illumina HiSeq platforms at the Lactad Facility (University of Campinas, Brazil). RNAseq datasets are available at NIH SRA (SRP158580).

We employed the GEMtools pipeline (https://github.com/gemtools/gemtools) of the GEM mapper [36] to align 128,411,588 paired-end reads sequenced from two replicates (76,963,784 vs. 51,447,804 reads in each of the libraries *vit1* and *vit2*) of the ovary samples to the *Rhodnius* genome assembly RproC1, using the subsequently quantified transcriptome annotation RProC1.1 as a guide. Overall ~90% (89.1 vs. 89.6%) of these reads mapped, and ~80% (80.9% vs. 79.2%) of the in total sequenced reads were considered informative for the quantification at a proportion of multi-mappings of <1%. These mappings exhibited a fidelity of on average ~3 mismatches and indels (3.2 respectively 3.5) with the RproC1 reference genome sequence and were used for subsequent quantification of the RproC1.1 transcriptome as annotated by the Vectorbase community (PMID: 22135296) and obtained from the Ensembl Metazoa database (v88). The EnsMart annotation of this database to maps 1,029 of the 1,467 most abundant mRNAs bidirectionally (i.e., orthology type "one-to-one") to protein-coding loci of the Flybase RefSeq annotation [37], further 208 Flybase proteins can be rescued through "one-to-many" and "many-to-many" ortholog mappings. After evaluating the number of occurrences for each term in comparison to their occurrence in the entire Flybase reference annotation, a p-value for the statistical overrepresentation is computed according to the model implemented in the DAVID tool [38]. Based on the distribution of p-values, we control the rate of false-discoveries to be not higher than 0.05 and group the remaining terms by a fuzzy clustering procedure on their co-occurrence in gene products, calling clusters with at least 5 members. The RproC1 version of the *Rhodnius* genome is available at the following URL: https://www.ebi.ac.uk/ena/data/view/GCA_000181055.2.

## Results

### Transcriptomic profiling of previtellogenic stages of oogenesis in *Rhodnius prolixus*

**Fig 1:**
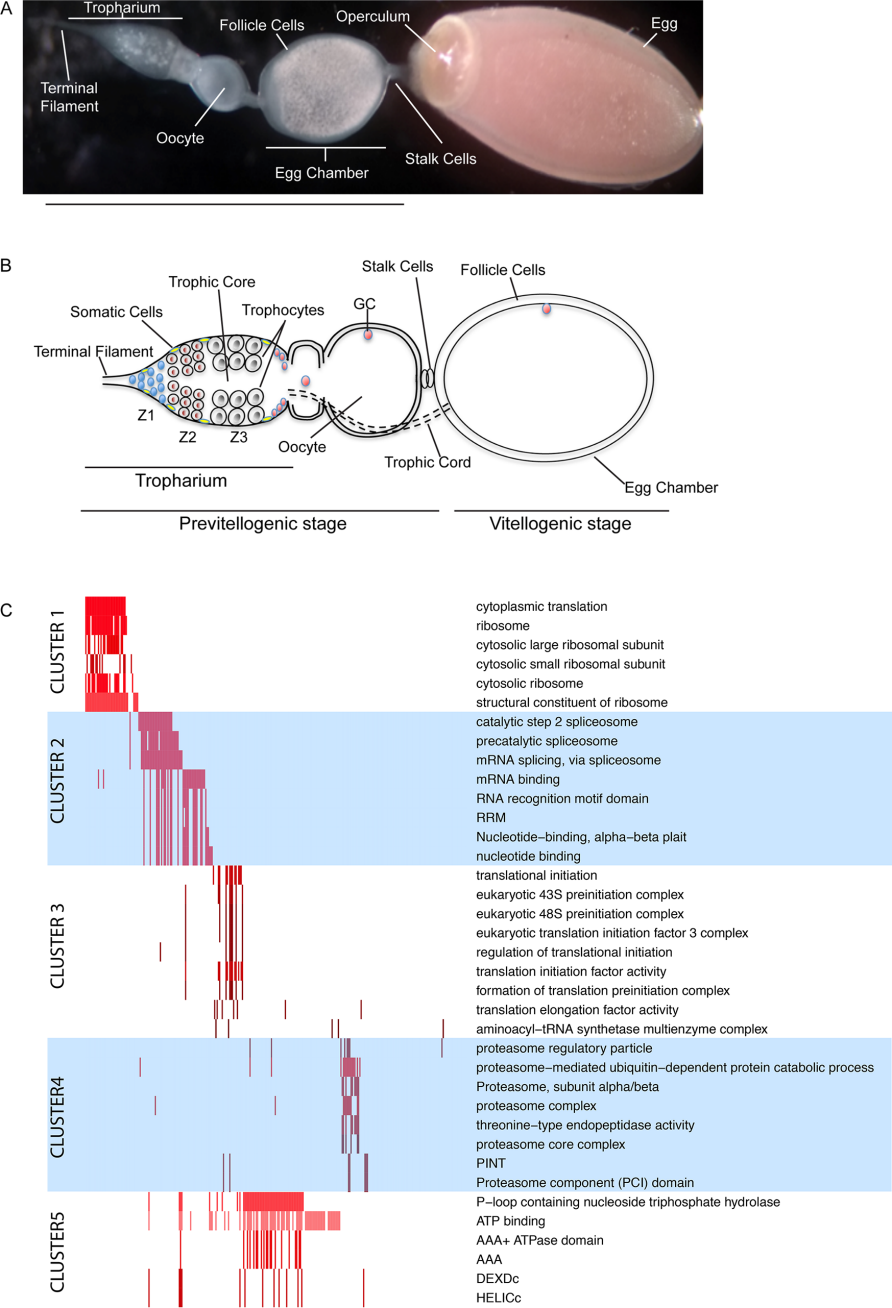
Transcriptomic analyses in early stages of *Rhodnius prolixus’* oogenesis. A) Structure of the ovariole showing the tropharium at the very anterior region, egg chambers in various stages of development and a mature chorionated egg at the very posterior end. The operculum defines the anterior pole of the egg. B) Schematic of the ovariole. The tropharium hosts mitotically active cells in zone 1 at the anterior region and the polyploid trophocytes (i.e. nurse cells) in zone 2 and 3. Oocytes arrested in Meiosis I are located at the posterior region of the tropharium. Each oocyte becomes encapsulated by follicle cells to form the budding egg chamber. Egg chambers in *Rhodnius* remain connected to the tropharium through the trophic cords, which ensure that nutrients and RNAs produced by the trophocytes are transferred to the growing oocytes. Z1, Zone1, Z2, Zone2, Z3, Zone3, GC, germinal vesicle. C) Functional Analysis of the previtellogenic transcriptome: Five major functional clusters (left) are identified for the 1,050 most expressed genes with high confident homologues in *D*. *melanogaster*, obtained by grouping statistically overrepresented terms (right) by their co-occurrence in gene products (x-axis). The matrix in the middle panel visualizes by colors the significance of the p-value computed for a gene.

Adult *Rhodnius* females develop two ovaries, each composed of groups of seven ovarioles (Fig 1A and 1B) [39–41]. Germline stem cells are present in nymphal stages and the adult females inherit a discrete number of oocyte arrested in meiosis I and aligned at the anterior region of a lancet-like structure termed the tropharium. During oogenesis, each oocyte is surrounded by a layer of somatic follicle cells to form the mature egg chamber. Different from the meroistic polytrophic ovary found in *Drosophila* and other species, in the meroistic telotrophic ovary of *Rhodnius* the egg chambers do not harbor nurse cells. Instead, the nurse cells or trophocytes populate the tropharium, where they form a syncytium around a central region termed the trophic core (Fig 1B). The tropharium can be divided in three regions with typical cell populations (Fig 1A and 1B). Actively dividing germ cells are observed only in the Zone1 at the very anterior region [42]. These cells migrate to the Zone2, where they lose their proliferation ability, begin the endoreduplication program and turn into trophocytes (Ts), which are functionally analogous to the *Drosophila* nurse cells. In the Zone3, that anticipates the previtellogenic egg chambers, the Ts display large nuclei with prominent nucleoli. In this region, the cell also start to degenerate and are progressively lost and replaced by new Ts migrating from Zone2. Nutrients and possibly RNAs produced by the trophocytes accumulate in the trophic core and are subsequently transported to the growing oocytes through specialized cytoplasmic bridges termed trophic cords. The previtellogenic phase of oogenesis starts in the tropharium and ends when the egg chambers reach a diameter of 0.5mm [43]. During vitellogenesis, the egg chambers grow up to 1mm in length, the trophic cords are severed and choriogenesis begins. Mature eggs produced by *Rhodnius* adult females are protected by a compact and resistant chorion, which regulates the fertilization process and prevents dehydration (Fig 1A). To investigate the molecular mechanisms that coordinate and drive *Rhodnius* oogenesis, we performed transcriptomic profiling in ovaries of blood-fed females. Our study focused on the previtellogenic phase of oogenesis. Total RNA extracted from these tissues was used to prepare and sequence paired-end RNAseq libraries and in total 12.84 Gigabases were sequenced in two replicates (vit1 and vit2). Sequence reads were then mapped to the *Rhodnius* genome RproC1 (Methods) as obtained from the Ensembl Metazoa database (v83) [41]. On average ~86% of informative mappings to the genome (55,455,772 in *vit1*, and 36,324,980 in *vit2*) superimposed in the correct orientation to the RProC1.3 transcriptome annotation. These mappings provide a deep interrogation of the annotated *R*. *prolixus* transcripts, with ~84% of the 14,840 transcripts detected by >10 read mappings, ~67% by >100 mappings, ~48% by >1,000 mappings, and ~10% by 10,000 mappings. Besides six rRNA loci (RPRC015844, RPRC015846, RPRC016406, RPRC016579, RPRC016706 and RPRC016876), these highly expressed loci comprise the RNAseP (RPRC016972) and two SRP genes (RPRC017200 and RPRC017302). Interestingly, we also found a putative ortholog of the *Drosophila squid* gene to be highly expressed in *Rhodnius* ovaries. The Squid protein controls the localization and translation of the *gurken* mRNA, which encodes a TGFa-like morphogen involved in the axial polarization of the egg and future embryo. Of the remaining 7,172 highly expressed protein-coding (pseudo-) genes, we investigated the 1,467 genes that exhibited > 10,000 reads for common functional patterns. Since only 36 of these were annotated with known protein functions in the RproC1.3 genome version, we employed for our functional study 9,188 orthology mappings to the *Drosophila melanogaster* proteome. Five major groups obtained by clustering 626 functional terms are obtained from these most abundant mRNAs (Fig 1C). These have been annotated in Flybase orthologs, collected from different databases and integrated into the DAVID functional annotation platform [44,45]. Our analysis reveals that genes with high expression levels in *Rhodnius* ovaries are orthologs of *Drosophila* proteins annotated with functions related to the ribosome (group 1) and translation (group 3), to mRNA processing and splicing (group 2), to proteasome activity (group 4), and also to helicases that pave the way for transcription of genes and ATP metabolism (group 5). In agreement with our samples being depleted of vitellogenic and choriogenic egg chambers, the functional classes related to vitellogenin biogenesis and uptake as well as chorion synthesis displayed low expression levels in our datasets. Our results demonstrate that cells in the previtellogenic phase of *Rhodnius* oogenesis invest the major part of their energy in the biogenesis (i.e., at the level of transcription, mRNA processing and translation) and turnover of the existing proteome by elevated proteome activity.

### Orthologs of the *Drosophila* piRNA pathway components are expressed during *Rhodnius* oogenesis

**Fig 2:**
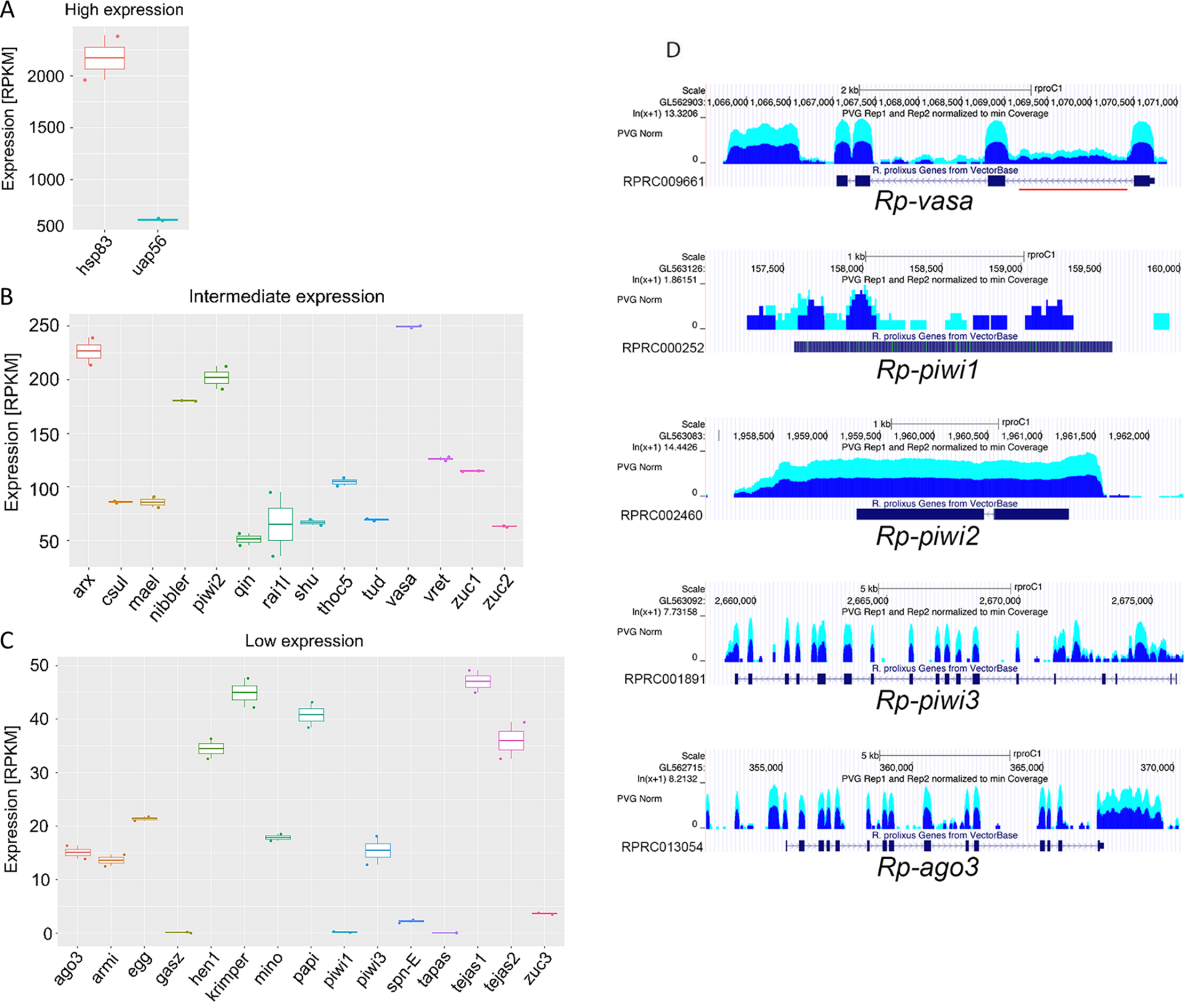
Steady-state expression levels of putative piRNA pathway genes in early *Rhodnius* oogenesis. A) Box plot showing the highly expressed putative orthologs of the fruit fly *uap56* and *hsp83* genes in *Rhodnius*. B) Box plot displaying 14 genes with intermediate expression levels in *Rhodnius* oogenesis. C) Box plots showing 15 genes, including putative orthologs of *spn-E* and *Rp-piwi1*, characterized by low expression levels in *Rhodnius*. In the panels A-C, Y-axis represents average RPKM values over two biological replicates. D) RNAseq profiles along the *Rp-vas*, *Rp-piwi1*, *Rp-piwi2*, *Rp-piwi3* and *Rp-ago3* genes. The Y-axis displays RPKM for two biological replicates (dark and light blue). Red line indicates the position of a *mariner*-like element in the *Rp-vas* gene. PVG stands for previtellogenic.

We employed our transcriptome profiling to determine the extent of evolutionary and functional conservation of the piRNA pathway in *R*. *prolixus*. Using Blast tools, we interrogated the Vectorbase platform to identify genes with homology to the *Drosophila* factors involved in the biogenesis and function of the piRNAs. For each putative ortholog, we then computed the expression levels as per RNAseq, as average RPKM between two biological replicates (Fig 2). We immediately noticed that the *heat shock protein 83* (*hsp83*) and *uap56* gene, which encode a nuclear-cytoplasmic RNA export factor, are expressed at higher levels (>500 RPKM) than other piRNA pathway components in *Rhodnius* ovaries (Fig 2A). The majority of the putative piRNA pathway genes however could be grouped in two classes: intermediate and low expression levels (Fig 2B and 2C). The first group is composed of 14 genes, whose steady state expression levels ranged between 50 and 250 RPKM (Fig 2B). These genes encode putative orthologs of several cytoplasmic factors involved in the biogenesis of the piRNAs in *Drosophila*, including Vas, Tudor, Maelstrom, and two putative orthologs of the Zuc endonuclease belong to this class. The remaining 15 genes displayed average RPKM lower than 50 (Fig 2C). Among them, *armitage*, the dSetDB1 methyl-transferase encoding *eggless* gene and the putative orthologs of *krimper*, *papi* and *tejas*, which encode Tudor domain proteins. Surprisingly, *spn-E*, a critical Helicase for the production of piRNAs in *Drosophila*, as well as the *piwi* ortholog *Rp-piwi1* seem to be either expressed at very modest levels or not expressed in *Rhodnius* ovaries (average RPKM<5) (Fig 2C). The results of the RNAseq analysis were validated by qRT-PCR assays with oligonucleotides specific to a selected group of genes (S2 Fig). In the fruit fly, the expression of the piRNA clusters in germline tissues is regulated by the RDC complex, which consists of the HP1 variant Rhino (Rhi), the Rai1-like factor Cutoff (Cuff) and the Deadlock (Del) protein. Blast alignments of the Rhi aminoacid sequence with proteins encoded in the *Rhodnius* genome did not return a clear match. Since this protein is a member of the Heterochromatin Protein (HP) family, several putative *Rhodnius* HP proteins share comparable aminoacid sequence similarity with Rhi. Conversely, the Del protein appears to evolve rapidly and is restricted to the Drosophilids. In *Drosophila*, the *cuff* and the CG9125 genes code for proteins with aminoacid sequence similarity to the yeast transcription co-factor Rai1. Interestingly, blast search analyses retrieve one single gene in *Rhodnius* encoding a putative protein displaying 20.7% and 28.1% aminoacid sequence identity with Cuff and with the protein encoded by CG9125 respectively. The *Rhodnius* Rai1-like gene, which we named *Rp-rai1l*, is not annotated in the current version of the *Rhodnius* genome and lies within the first intron of the gene RPRC008241 (supercontig KQ034693) (S1 Fig). Our transcriptomic analysis reveals that *Rp-rai1l* displays intermediate expression levels during *Rhodnius* oogenesis (Fig 2B), although its role, if any, in the piRNA pathway needs to be elucidated. For several genes that have been linked to the piRNA pathway in *Drosophila*, our blast search did not return homologous sequences in the *Rhodnius* genome. For instance, the *Rhodnius* genome does not appear to host homologs of the *Drosophila moon* and *squash* genes, which are expressed in the germline tissues, as well as of *YB*, *SoYB*, *BoYB* and *T2RD2* that act in the somatic branch of the piRNA pathway in the fruit fly.

We then focused our study on the *Rhodnius* orthologs of the *vas* and *piwi* genes, which are central components of the piRNA pathway in *Drosophila*. Vas is a DEAD-box RNA helicase related to the translation factor eIF-4A and has been extensively used as a marker of germline tissues in distantly related organisms [46]. Importantly, studies in *Drosophila* showed that Vas elicits piRNA production in concert with Aub and Ago3 [16]. We found that the *Rhodnius* genome harbors a putative homolog of the *vas* gene (Vectorbase ID RPRC009661), which encodes a protein 75% identical to DmVas. Our RNAseq profiling reveals that *Rp-vas* is expressed at intermediate levels during *Rhodnius* oogenesis (Fig 2B). Interestingly, while the *D*. *melanogaster vas* hosts the *vig* and *solo* genes in its intronic sequences, this arrangement is absent in *Rhodnius*, where the *Rp-vas* intron 1 harbors an Open Reading Frame (ORF) encoding a transposase enzyme from a *mariner*-like element (Fig 2D). Transcripts of this ORF are readily detected in our ovarian transcriptome datasets. Previous studies reported that *Rhodnius* displays an amplification of the *piwi* genes, whereby three putative orthologs of *piwi*, namely *Rp-piwi1*, *Rp-piwi2* and *Rp-piwi3*, in addition to the *Rp-ago3* ortholog of the *Drosophila ago3* gene are present in the genome [26]. The analysis of the normalized RNAseq reads shows that *Rp-piwi2*, *Rp-piwi3* and *Rp-ago3* are expressed at intermediate or low levels in *Rhodnius* oogenesis (Fig 2B–2D), while the *Rp-piwi1* transcripts are barely detectable. Based on the absence of intronic sequences, it has been proposed that *Rp-piwi1* is a pseudogene and might not be required for *Rhodnius* development. Our transcriptomic analyses seem to support this hypothesis, although we cannot rule out the *Rp-piwi1* might be expressed in tissues other than the ovary. According to the current genome annotation, *Rp-piwi2* contains a small intron of approximately 100bp. Our RNAseq analysis however reveals that this sequence is included in the mature transcript and the resulting ORF encodes a putative protein of 882aa. Furthermore, the 5' and 3' untranslated regions of the *Rp-vas*, *Rp-piwi2*, *Rp-piwi3* and *Rp-ago3* extend beyond the limits annotated in the current version of the genome. Thus, our datasets not only provide information on the steady-state expression levels for all the genes and loci expressed in previtellogenic stages of *Rhodnius* oogenesis, but will also contribute to improve gene annotation and discovery.

### Evolutionary conservation of the putative Rp-PIWI proteins

**Fig 3:**
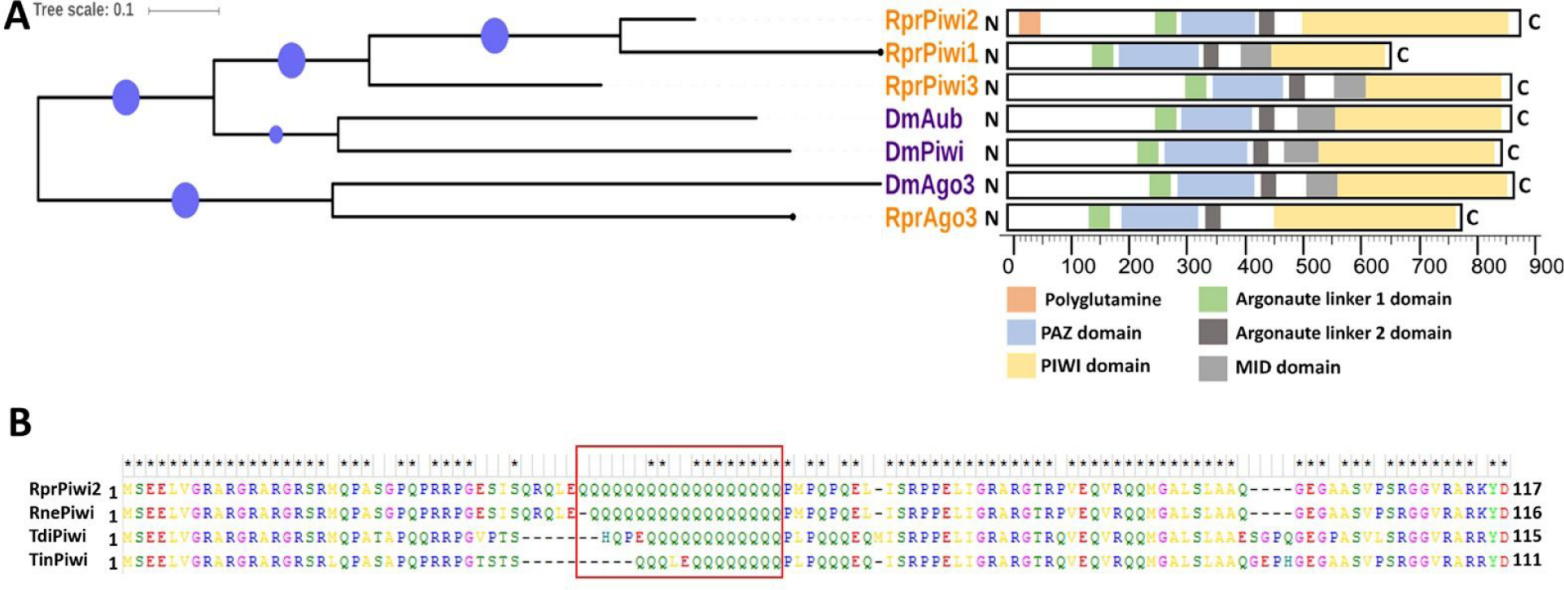
Evolutionary conservation of the putative PIWI proteins in *Rhodnius*. A) Phylogenetic tree displaying the evolutionary relationship among Rp-Piwi1, Rp-Piwi2, Rp-Piwi3 and Rp-Ago3 and their *Drosophila* orthologs Piwi, Aub and Ago3. Schematic displaying the conservation of the typical Argonaute domains in the *Drosophila* and *Rhodnius* Piwi-clade Argonaute proteins. The conserved Piwi (yellow), PAZ (blue) and MID (grey) domains are highlighted. Argonaute linker domains 1 (green) and 2 (dark grey) are also shown. A polyglutamine stretch (orange) is present at the N-terminal region of the putative Rp-Piwi2 protein. B) Multiple aminoacid sequence alignment of the N-terminal regions of the putative Rp-Piwi2 orthologs in *Rhodnius prolixus*, *Rhodnius neglectus*, *Triatoma dimidiata* and *Triatoma infestans*. The red box highlights the position of the conserved PolyQ tract in the different Piwi orthologs.

The *Rp-piwi1*, *Rp-piwi2* and *Rp-piwi3* (VectorBase IDs RPRC00252, RPRC002460 and RPR001891) encode putative proteins with 38.7%, 36.1% and 43.3% aminoacid sequence identity with *D*. *melanogaster* Piwi respectively (Fig 3A and S2 Fig). While the *piwi* orthologs appear to have originated from duplication of an ancestral *piwi* gene, the *Rp-ago3* gene is homologous to the *Drosophila ago3* gene (Fig 3A and S2 Fig). Accordingly, the *Rp-ago3* locus (Vectorbase ID RPRC013054) encodes a putative protein 43.3% identical to DmAgo3. We then analyzed the degree of sequence identity between the individual Piwi, MID and Paz domains across the PIWI proteins in *Drosophila* and *Rhodnius* (Fig 3A and S2 Fig). All the domains appear to be well conserved in all the *Rhodnius* orthologs including the putative PIWI protein encoded by the *Rp-piwi1* gene. Interestingly, the N-terminal region of the putative Rp-Piwi2 protein features a stretch of 18 Glutamine residues (i.e. polyQ tract) (Fig 3A and S2 Fig). This characteristic has not been reported for any of the PIWI proteins so far analyzed in a range of animal species. We therefore wondered whether PolyQ stretches are present in PIWI related proteins from other insects. Using NCBI Blast search analyses, we found that the polyQ tract is present in Piwi-like proteins encoded in the genome of *Rhodnius neglectus*, *Triatoma infestans* and *Triatoma dimidiata* (Fig 3B), but it is absent in PIWI proteins of *Drosophila* and other animals (Fig 3A and S2 Fig). Thus, the acquisition of a PolyQ sequence is likely a recent evolutionary event and is restricted to certain Triatomine species.

### Expression pattern of the *Rp-vas* and *Rp-piwi* genes during previtellogenesis

**Fig 4:**
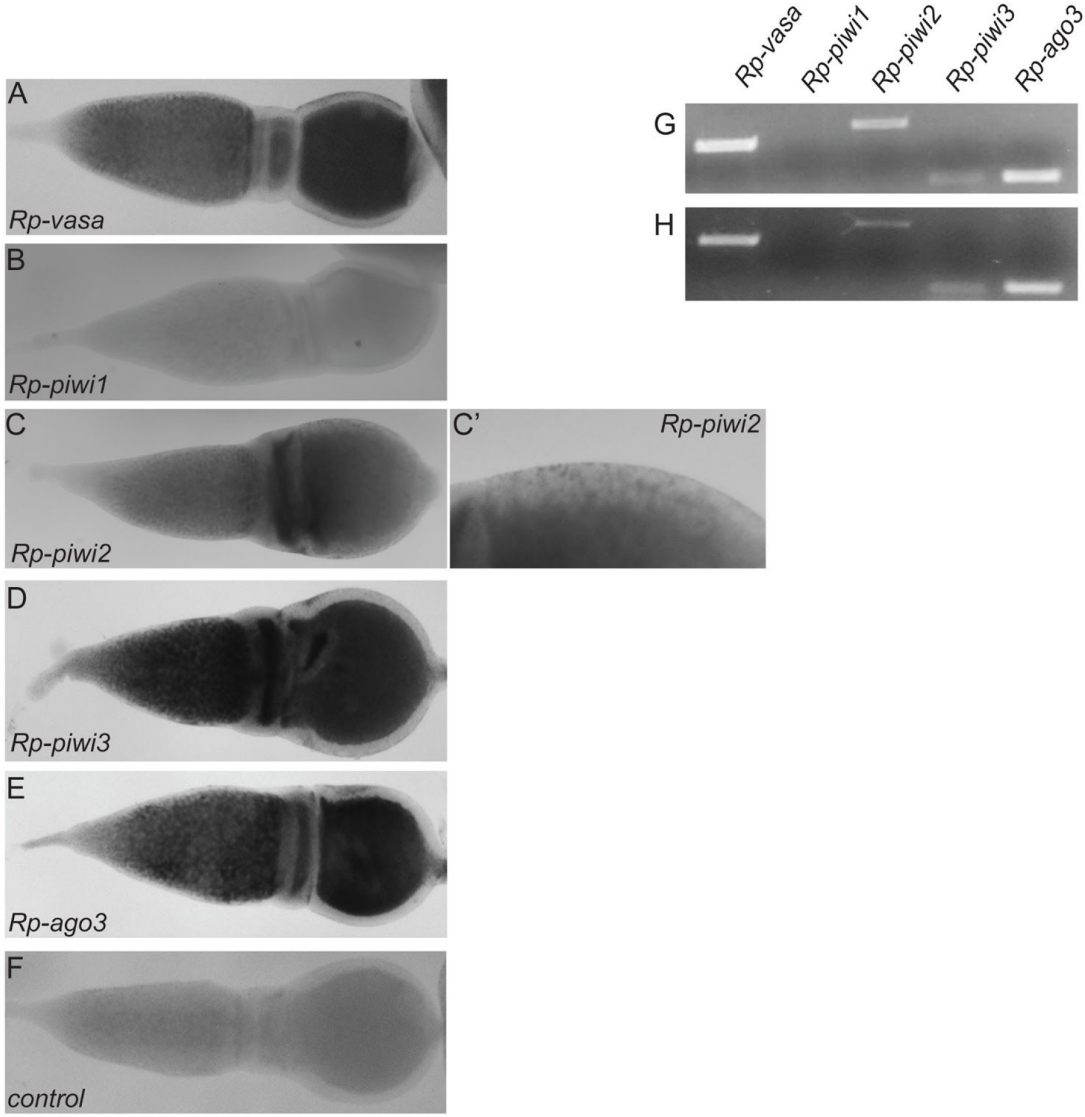
Expression pattern of the *vas* and *piwi* orthologs in *Rhodnius*. A) *In situ* hybridization assays in early stages of oogenesis (tropharium and vitellarium) for *Rp-vas* (panel A), *Rp-piwi1* (panel B), *Rp-piwi2* (panels C and C'), *Rp-piwi3* (panel D) and *Rp-ago3* (panel E) genes. A sense *Rp-ago3* probe was used as control for these assays (panel F). G-H) RT-PCR in previtellogenic stages of oogenesis (panel G) and in mature eggs (panel H) with oligonucleotides specific to *Rp-vas*, *Rp-piwi1*, *Rp-piwi2*, *Rp-piwi3* and *Rp-ago3* genes.

Next, we wondered whether the *Rp-vas* and the *Rp-piwi* genes display stage- or tissue-specific expression patterns during *Rhodnius* oogenesis. To answer this question, we performed *in situ* hybridization assays in fixed ovaries using antisense probes corresponding to specific sequences within the ORF of *Rp-vas*, *Rp-piwi1*, *Rp-piwi2*, *Rp-piwi3* and *Rp-ago3* (Fig 4). This approach revealed that the expression of *Rp-vas* is restricted to the germline tissues given that the *Rp-vas* probe generates a signal in the tropharium and in the oocyte, but not in the somatic follicle cells (Fig 4A). Thus, RPRC009661/*Rp-vas* encodes a bona fide ortholog of DmVas, which allows distinguish the germ cell lineage from the somatic cell population. Previous studies reported the expression of a putative *vas* ortholog in the somatic follicle cells of the *Rhodnius* ovary [47]. Our *in situ* hybridization protocols did not allow preserve the vitellogenic egg chambers, thus we could not determine whether RPRC009661/*Rp-vas* transcripts are produced in the follicular epithelium in late stages of oogenesis. In accordance with the RNAseq data, the *Rp-piwi1* probe did not produce any signals above the background levels (Fig 4B). The *Rp-piwi2* transcripts instead are clearly detected in the tropharium and in the developing oocytes (Fig 4C). Interestingly, the *Rp-piwi2* RNAs seem to unevenly accumulate in more mature oocytes, where they are enriched in the anterior region (Fig 4C). In addition, the *Rp-piwi2* transcripts are detected also in the somatic follicle cells, thus suggesting that the expression of this gene is not restricted to the germline tissues (Fig 4C'). Similar to *Rp-vas*, the *Rp-piwi3* and *Rp-ago3* transcripts are detected in the region of the tropharium hosting the polyploid Ts and in the ooplasm of newly formed and mature egg chambers, but not in the follicle cells (Fig 4D and 4E). As control assay, we generated a sense probe corresponding to a region of the *Rp-ago3* ORF (Fig 4F). This probe did not produce specific signals above the background levels.

During *Rhodnius* oogenesis, RNAs and nutrients produced by the nurse cells hosted in the tropharium are transported to the growing oocytes through the trophic cords. In order to determine whether *piwi* and *vas* transcripts are maternally stored in the mature eggs, we performed RT-PCR assays in ovaries with oligonucleotides specific for the *piwi* and *vas* genes by separating the previtellogenic stages of oogenesis from the chorionated mature eggs. In both stages of *Rhodnius* oogenesis, a clear amplification product of the expected molecular weight was detected for the *Rp-piwi2*, *Rp-piwi3*, *Rp-ago3* and *Rp-vas* genes, while no amplification signal was produced for *Rp-piwi1* (Fig 4G and 4H). These observations point to a direct role for *Rp-piwi2*, *Rp-piwi3*, *Rp-ago3* and *Rp-vas* in *Rhodnius* germline development and possibly early embryogenesis.

### *Rp-piwi2*, *Rp-piwi3* and *Rp-ago3* ensure female adult fertility in *Rhodnius*

**Fig 5:**
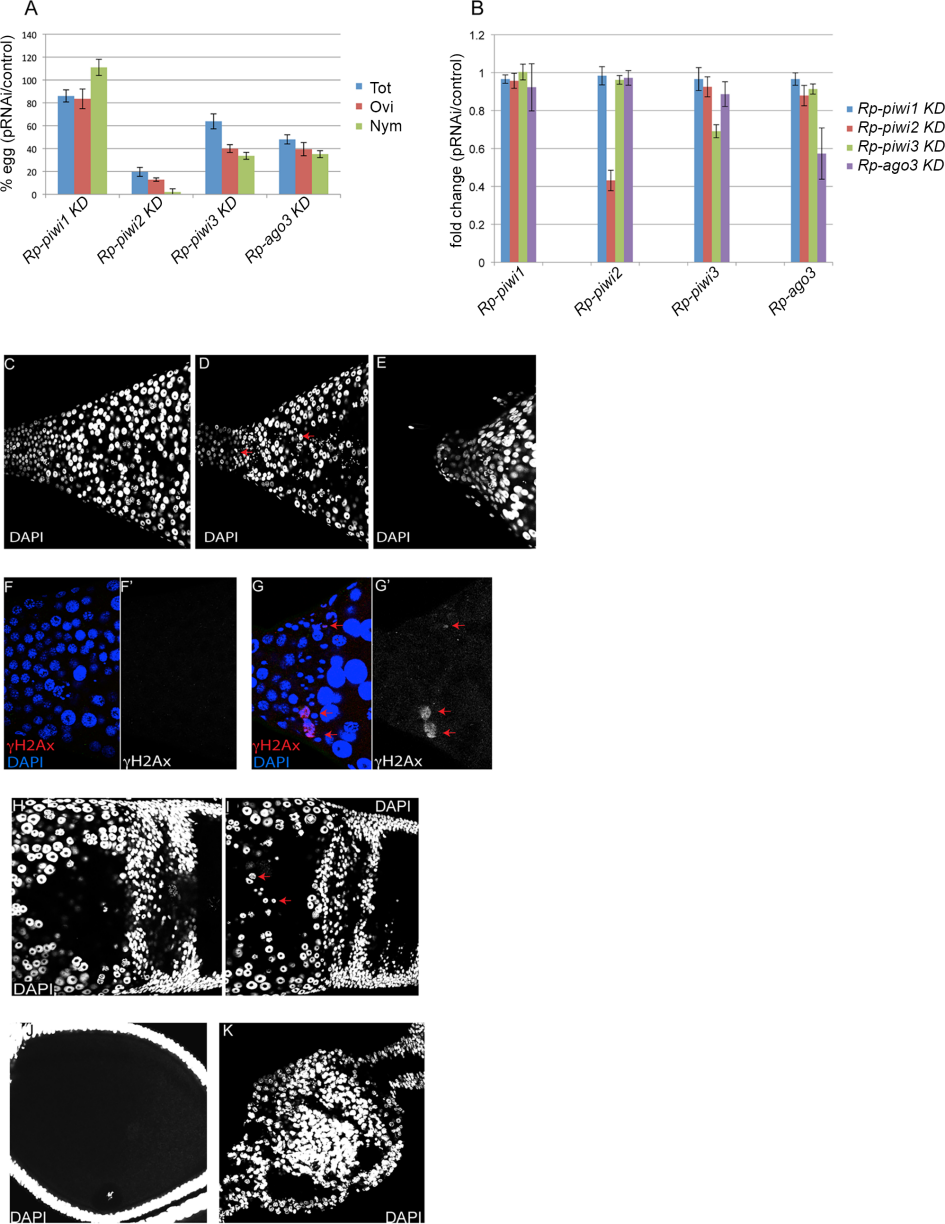
Functional analysis of the Rp-piwi genes. A) Parental RNAi against the *piwi* orthologs. Oogenesis and fertility were investigated by dividing the eggs in three groups: total number of eggs (Tot), oviposited eggs (Ovi) and hatched eggs giving rise to first-instar nymphs (Nym). Y-axis displays the percentage eggs produced by *Rp-piwi* pRNAi treated females compared to control pRNAi females. B) qRT-PCR assays to investigate the expression levels of the *Rp-piwi* genes after pRNAi treatment. The Y-axis displays the fold difference in the expression levels of the *Rp-piwi1*, *Rp-piwi2*, *Rp-piwi3* and *Rp-ago3* in each of the *Rp-piwi* KD versus control-injected ovaries. Error bars indicate standard deviation. C-E) Ovarian phenotype of *Rp-piwi2* pRNAi females. Zone1 of control (C) and *Rp-piwi2* pRNAi (D) ovaries. pRNAi against the *Rp-piwi2* gene leads to a reduction in the number of trophocytes and abundant DAPI-positive nuclear debris (red arrows). (E) Zone 1 of *Rp-piwi2* pRNAi ovaries is frequently atrophic. F-G) Immunostaining with anti-γH2Ax antibodies (red) and DAPI (blue) in Zone1 of control (F) and *Rp-piwi2* pRNAi (G) ovaries. Notice the degenerating nuclei and DAPI-positive particles (red arrow). Single channels for the γH2Ax signal in control (F’) and pRNAi (G’) are shown. H-I) Zone3 and previtellogenic egg chambers of control (H) and *Rp-piwi2* pRNAi (I) ovaries stained with DAPI. Degenerating nuclei appear to accumulate in the trophic core of *Rp-piwi2* KD tropharia (red arrows). J-K) Control and *Rp-piwi2* pRNAi egg chambers. Notice the cortical position of the germinal vesicle and the organized layer of follicle cells surrounding the oocyte in the control (J) ovaries, while egg chambers appear collapsed or atrophic upon pRNAi for *Rp-piwi2* (K).

Parental RNAi (pRNAi) was previously shown to induce the efficient reduction of gene expression in *Rhodnius*, where genetic tools are still lacking [48]. In order to understand the function of the *Rp-piwi* genes in *Rhodnius*, we carried on pRNAi assays by injecting dsRNA molecules targeting portions of their coding regions in the abdomen of adult females (Fig 5A). Injected females were blood-fed, their eggs were collected daily over a period of three weeks and let to develop until the first-instar nymphs emerged. After the 3-weeks period, the females were dissected and the eggs retained in the abdomen were also counted. This approach allowed us to investigate the oviposition and fertility of the injected females. Eggs were divided into three bins: 1) total number of eggs, which is given by the sum of the eggs retained in the abdomen and those that were actually oviposited, 2) oviposited eggs, 3) eggs that hatched to produce first-instar nymphs. Each group of control-injected females produced on average a total of 262 eggs of which 103 were oviposited and 67 developed into first-instar nymphs. Compared to these control animals, the *Rp-piwi1* pRNAi females produced on average a slightly lower number of total eggs (~86%) and oviposited eggs (~83%). However, the hatching rates were higher than the control (~111%). It is noteworthy that in our pRNAi assays, the Rp-*piwi1*-KD females consistently produced a slightly higher number of first-instar nymphs than the control females, although this gene is apparently not expressed in ovaries. In contrast, oogenesis seemed to be partially impaired by pRNAi-mediated downregulation of the *Rp-piwi3* and *Rp-ago3* genes. Compared to the control, females from these assays respectively produced ~63% and ~48% total eggs, ~40% and ~39% deposited eggs and ~33% and ~35% first-instar nymphs. The most striking result however was obtained upon injection of *Rp-piwi2* dsRNA molecules in adult females. The total number of eggs and the number of oviposited eggs produced on average by these animals was ~19% and ~13% of the control values, respectively. More importantly, the first-instar nymphs generated by *Rp-piwi2* pRNAi females were only ~2% of the control values.

In order to investigate the specificity of our dsRNAs for the respective cognate *Rp-piwi* gene, we performed qRT-PCR assays in previtellogenic stages of pRNAi ovaries with oligonucleotides specific to *Rp-piwi1*, *Rp-piwi2*, *Rp-piwi3* and *Rp-ago3* (Fig 5B). As internal control for this assay, we used oligonucleotides specific to *Rp-rp49* (RPRC014419), a putative *Rhodnius* ortholog of the *Drosophila rp49* gene. These assays revealed that each dsRNA specifically downregulates the expression of the cognate gene by ~30% for *Rp-piwi3*, ~40% for *Rp-ago3* and >50% for *Rp-piwi2*. As expected, the injection of *Rp-piwi1* dsRNA did not produce significant changes in the expression levels of *Rp-piwi1*. Our results strongly point to a critical role for *Rp-piwi2*, *Rp-piwi3* and *Rp-ago3* in *Rhodnius* oogenesis and female adult fertility.

### The *Rp-piwi2* gene controls germ cell survival and egg chamber development during *Rhodnius* oogenesis

We then sought to determine the cellular basis of the reduced fertility observed in *Rp-piwi2*, *Rp-piwi3* and *Rp-ago3* KD females. To this aim, we immunostained ovaries from pRNAi-injected adult females with DAPI to visualize the nuclei of the germ cells and of the follicle cells (Fig 5). We immediately noticed that *Rp-piwi2* pRNAi tropharia displayed abundant DAPI-positive particles in Zone1 and Zone2, which are rarely observed in the tropharia of control ovaries (Fig 5C and 5D). In some cases, the anterior tip of the tropharium corresponding to Zone1 appeared severely atrophic (Fig 5E). The γH2Ax histone variant was shown to accumulate at sites of DNA damage, induced for instance by meiotic recombination events or by transposable element mobilization [23,49]. We therefore monitored the occurrence of DSBs in control and *Rp-piwi2*-KD ovaries with the antibodies specific to γH2Ax (Fig 5F, 5F’, 5G and 5G’). This assay revealed that the DAPI particles observed in Zone1 of *Rp-piwi2-*KD tropharia as well as some nurse cell nuclei are enriched in this histone variant. In contrast, control tropharia did not display any signal beyond the background levels. In Zone 3 of control tropharia, the Ts nuclei are arranged at the periphery of the tropharium and display apparent nucleoli (Fig 5H). Instead, the Zone3 of the pRNAi-treated ovaries clearly displays a lower number of Ts nuclei and abundant nuclear debris in the trophic core (Fig 5I). In *Rhodnius*, each egg chamber is formed by an oocyte surrounded by a follicular epithelium (Fig 5J). pRNAi for *Rp-piwi2* seems to strongly impair the progression through oogenesis and the growth of the egg chambers. We frequently observed smaller and apparently collapsed egg chambers during vitellogenesis (Fig 5K). These atrophic egg chambers were still connected posteriorly to younger egg chambers emerging from the tropharium and anteriorly to more mature choriogenic egg chambers through bridges of stalk cells. Despite the clear impact of pRNAi on *Rhodnius* fertility, the analysis of *Rp-piwi3* and *Rp-ago3* pRNAi ovaries did not display obvious abnormalities by DAPI staining and anti-γH2Ax immunostainings and additional molecular tools will be necessary to dissect their function during germline development. Our results however, demonstrate that *Rp-piwi2* in fundamental for Ts survival and egg chamber development during *Rhodnius* oogenesis.

## Discussion

PIWI proteins complexed with piRNAs coordinate a defense system that represses mobile genetic elements and protects the genome of animal germ cells. In this study, we show that central components of the piRNA pathway, first described in *Drosophila* are conserved in the hemimetabolous insect *Rhodnius prolixus*, which is 350mya distant from the fruit fly. *Rhodnius* harbors four putative *piwi* genes, and we show that *Rp-piwi2*, *Rp-piwi3* and *Rp-ago3*, but not *Rp-piwi1*, are expressed in ovaries. In order to investigate their expression patterns during oogenesis, we first identified RPRC009661 as a *vas* ortholog in *Rhodnius* and we showed that it is a germline-specific gene. The *Rp-piwi3* and *Rp-ago3* transcripts display a germline-specific expression patterns similar to *Rp-vas* and appear enriched in the growing oocytes. Interestingly, *Rp-piwi2* transcripts can be detected both in the somatic as well as in the germ cells and seem to display an asymmetric localization pattern during oocyte development. This gene is expressed in the tropharium and its transcripts evenly accumulate in the budding egg chamber. In the neighboring and more mature egg chamber, however, *Rp-piwi2* transcripts are enriched at the anterior pole of the oocytes. Our *in situ* hybridization assays suggest that *Rp-piwi2* transcripts might diffuse from the ooplasm of the budding egg chamber into the neighboring more mature oocyte. Alternatively, *Rp-piwi2* expression might occur in the invading follicle cells that form the boundary between the budding egg chambers and the transcript deposited in the adjacent oocytes. It will be of great interest to determine whether the *Rp-piwi2* expression pattern impacts the axial polarization of the *Rhodnius* eggs and embryos. Our functional studies using pRNAi against the *Rp-piwi2* gene resulted in oogenesis arrest and complete female adult sterility. In wild type ovaries, the Zone 1 of the tropharium hosts mitotically active trophocytes, which replenish the population of polyploid Ts in Zone 2 and 3. Reduction of the *Rp-piwi2* levels by pRNAi causes a severe loss of dividing cells in Zone 1 and of polyploid Ts in Zone 2 and 3. The accumulation of γH2Ax-positive nuclear debris in the tropharia of injected females strongly suggests that Ts degenerate in *Rp-piwi2* KD ovaries. The loss of Ts in turn likely results in dumping phenotypes, which explain the oogenesis arrest and the frequently collapsed egg chambers observed in these females. It is tempting to speculate that the DNA damage and the loss of Ts observed in the *Rp-piwi2* KD ovaries might be caused by the deregulation of transposable elements. The percentage of transposable elements in the *Rhodnius* genome is approximately 6% and two thirds of the transposons in this species belong to the *mariner* family [41,50,51]. Rp-piwi2 might be required to silence these elements in the germline and, possibly, in the somatic tissues. The cloning and characterization of the piRNA population will be necessary to shed more light on the function of *Rp-piwi2* and the piRNA pathway in this species. Remarkably, we found that the putative Rp-Piwi2 protein features a 18aa Polyglutamine (PolyQ) tract at its N-terminal region. PolyQ repeats have been identified in various proteins of organisms as distant as plants and vertebrates and are often found in transcription factors. Interestingly, the PolyQ stretch appears to be conserved in putative PIWI proteins of the closely related species *Rhodnius neglectus*, *Triatoma infestans* and *Triatoma dimidiata*, while it is not present in the PIWI proteins of other organisms including *Drosophila*. Thus, the PolyQ tract is likely a recent acquisition in the evolution of the PIWI proteins and, based on the available sequenced genomes, appears to be restricted to blood-feeding insects of the Triatomine family.

Albeit to a lesser extent, *Rp-piwi3* and *Rp-ago3* KDs also affect egg production and female adult fertility in *Rhodnius*. The expression of *Rp-piwi1* gene instead is negligible in *Rhodnius* ovaries and *Rp-piwi1* dsRNA injection in adult females does not negatively affect oogenesis and fertility.

In addition to the *Rp-piwi* genes our transcriptomic analysis revealed that several components of the piRNA pathway are conserved and expressed in the ovary of this species. We did not find evidence of an RDC complex in *Rhodnius* except for a putative protein (i.e. Rp-Rai1l) with similarity to Cuff. If piRNA clusters exist in this species, it is likely that their regulation relies on a set of proteins different from the one described in *Drosophila*. However, we provide evidence that several factors involved in the transport and processing of the pre-piRNAs, including Uap56, Krimper and Maelstrom among others, are expressed during *Rhodnius* oogenesis. Yet, some critical germline factors, like the Helicase SpnE, are expressed at very low levels. Similarly, the somatic branch of the piRNA pathway might rely on the activity of the *Rp-piwi2* gene and the *zuc*, *armi* and *vret* orthologs, while YB, BoYB, SoYB and T2RD2, which associate with the YB bodies and catalyze the production of mature piRNAs in the *Drosophila* follicle cells, are not present in the *Rhodnius* genome. These genes have been reported to be absent also from the genome of other insect species, including the Honeybee *Apis mellifera* and *Tribolium castaneum* [52]. Thus, both branches of the piRNA pathway are partially conserved in insects and it will be a challenge for the future to fully understand the differences between *Drosophila* and *Rhodnius*.

*Rhodnius prolixus* together with other Triatomine species are major vectors of the protozoan *Trypanosoma cruzi*, the causal agent of the Chagas disease. In this study, we shed light on the ovarian transcriptome of *Rhodnius* and unveiled the degree of evolutionary and functional conservation of the piRNA pathway in this species. Furthermore, we show that *piwi* genes are essential for oogenesis and adult fertility in *Rhodnius* and likely exert similar functions in other Triatomine species. Sterile Insect Techniques (SIT) have been extensively used to reduce natural populations of insects of medical or economic importance in many countries [53].Thus, our results provide a framework for the development of novel strategies to control the natural populations of Triatomine insect vectors and reduce the spread of the Chagas disease.

## Supporting information

S1 Table List of genes expressed during early *Rhodnius* oogenesis as per RNAseq. For each gene, the genomic location, gene ID, putative orthologs in *D*. *melanogaster*, biotype and length are provided along with the raw number of aligned reads, Reads Per Million (RPM) and Reads Per Kilobase per Million (RPKM) for each biological replicate (rep1 and rep2). The average RPKM and Standard Deviation over the biological replicates are also listed.
(XLSX)

S2 Table List of oligonucleotides used in this study. (XLSX)

S1 Fig qRT-PCR analysis of the expression levels of 9 selected genes in previtellogenic stages of *Rhodnius* oogenesis. Y-axis displays the average Ct values for each gene over biological triplicates. Error bars indicate standard deviation.
(TIF)

S2 Fig Expression levels and genomic position of the *Rp-rai1l* gene. RNAseq profile along a region of the contig KQ034693 of the *Rhodnius* genome. The position of the *Rp-rai1l* gene is highlighted by dotted red lines.
(TIF)

S3 Fig Multiple aminoacid sequence alignment between the *Drosophila* and *Rhodnius* PIWI proteins. The conserved Ago N-terminal (light green), Ago Linker 1 (blue), PAZ (red), Ago Linker 2 (dark green), MID (yellow) and Piwi (light blue) are highlighted.
(TIF)
