# Supplementary figures and images for: Transcriptomic and functional analyses of the piRNA pathway in the Chagas disease vector Rhodnius prolixus
Source: PLoS Negl Trop Dis. 2018 Oct 10;12(10):e0006760. doi: 10.1371/journal.pntd.0006760 (PMC6179187; doi:10.1371/journal.pntd.0006760)

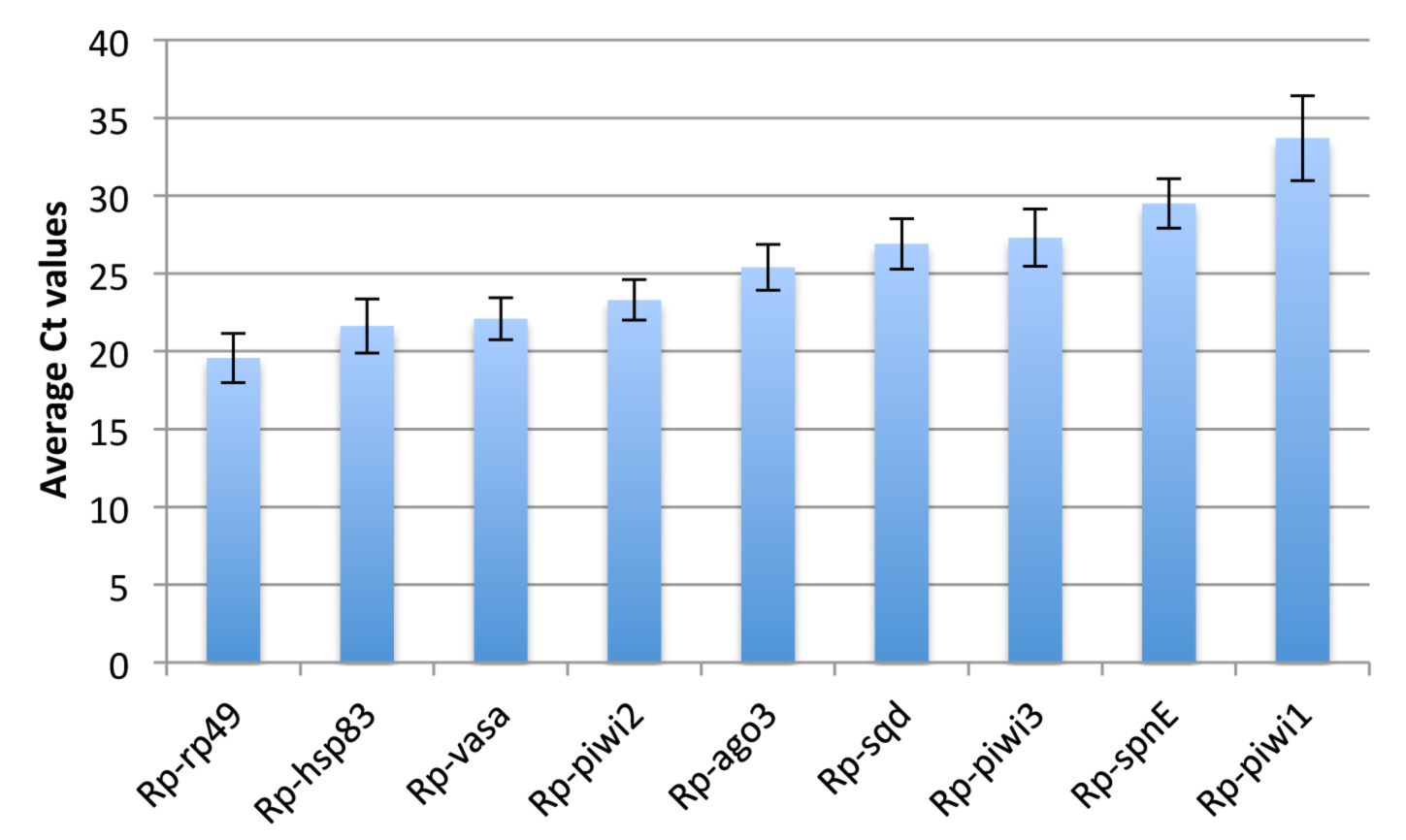

Supplement: S1 Fig — Y-axis displays the average Ct values for each gene over biological triplicates. Error bars indicate standard deviation. (TIF) [file pntd.0006760.s003.tif]

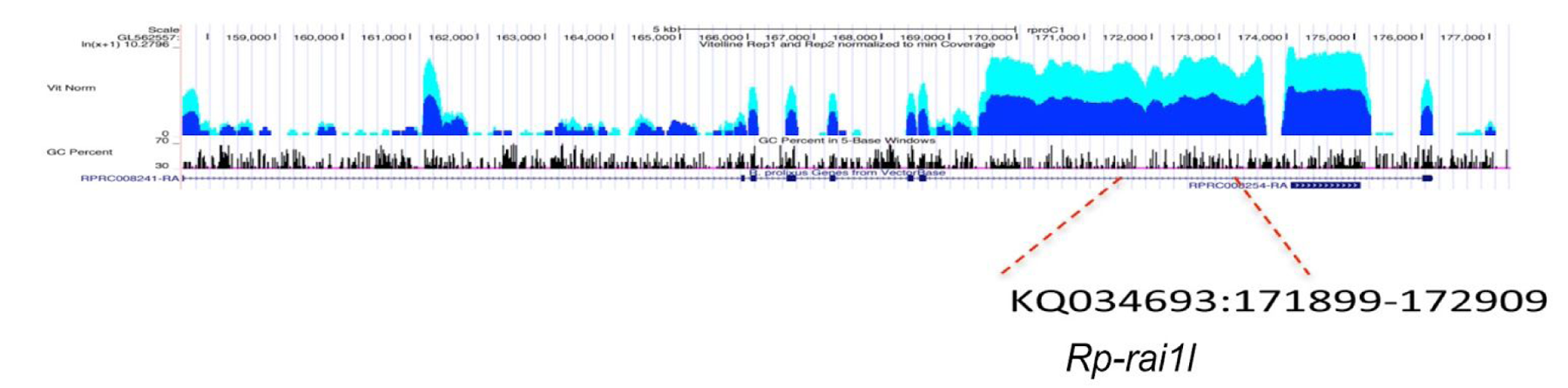

Supplement: S2 Fig — RNAseq profile along a region of the contig KQ034693 of the Rhodnius genome. The position of the Rp-rai1l gene is highlighted by dotted red lines. (TIF) [file pntd.0006760.s004.tif]

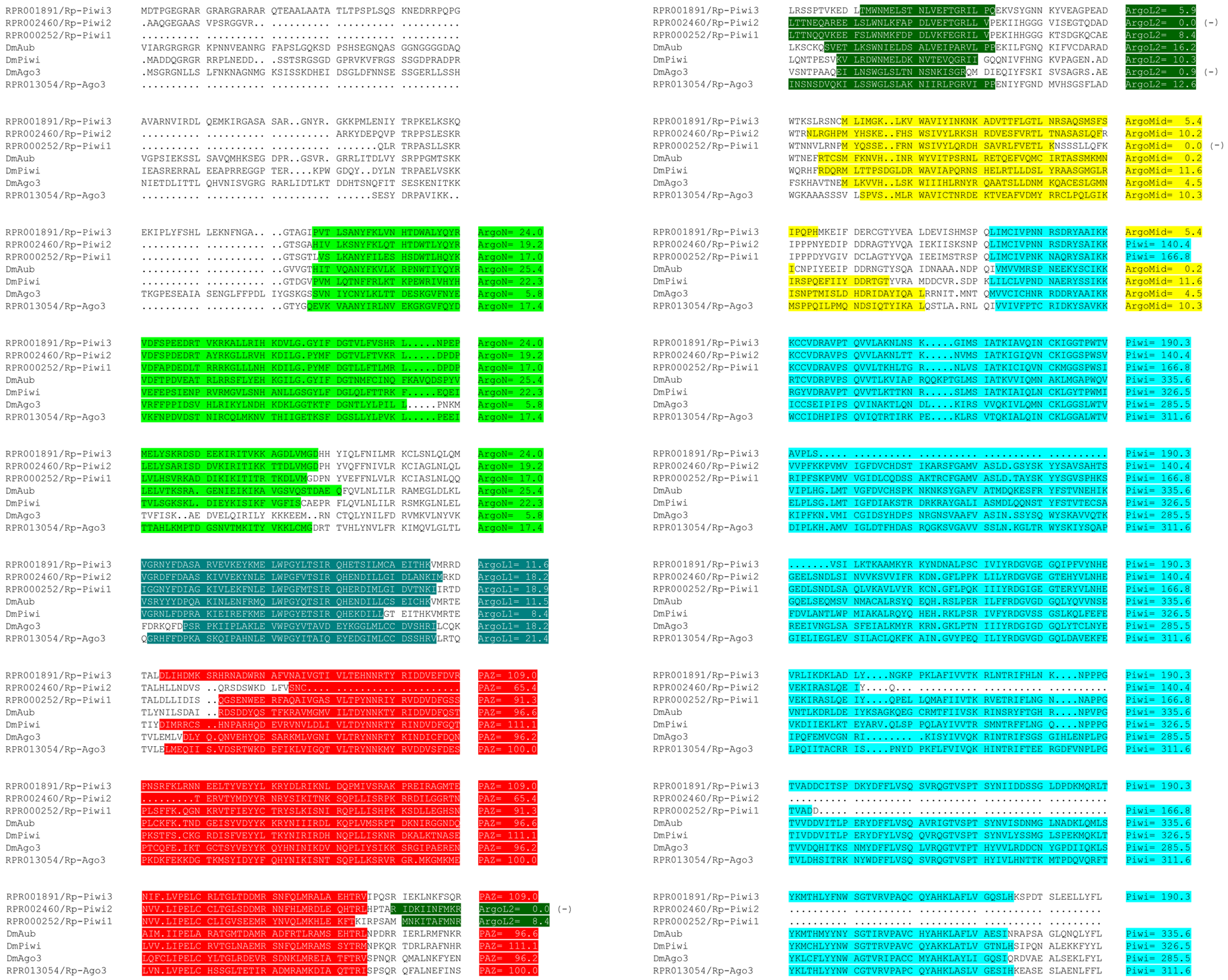

Supplement: S3 Fig — The conserved Ago N-terminal (light green), Ago Linker 1 (blue), PAZ (red), Ago Linker 2 (dark green), MID (yellow) and Piwi (light blue) are highlighted. (TIF) [file pntd.0006760.s005.tif]
